# Theorising women’s health and health inequalities: shaping processes of the ‘gender-biology nexus’

**DOI:** 10.1080/16549716.2019.1669353

**Published:** 2019-10-07

**Authors:** Ellen Annandale, Maria Wiklund, Anne Hammarström

**Affiliations:** a Department of Sociology, University of York, York, England; b Department of Community Medicine and Rehabilition, Physiotherapy, Umeå University, Umeå, Sweden; c Institute of Environmental Medicine, Karolinska Institutet, Stockholm, Sweden; d The Stress Research Institute, Stockholm University, Stockholm, Sweden

**Keywords:** Gender and Health Inequality, Biology, female genital mutilation/cutting, inequality, feminism, theoretical

## Abstract

Since the theoretical frameworks and conceptual tools we employ shape research outcomes by guiding research pathways, it is important that we subject them to ongoing critical reflection. A thoroughgoing analysis of the global production of women’s health inequality calls for a comprehensive theorization of how social relations of gender and the biological body mutually interact in local contexts in a nexus with women’s health. However, to date, the predominant concern of research has been to identify the biological effects of social relations of gender on the body, to the relative neglect of the co-constitutive role that these biological changes *themselves* may play in ongoing cycles of gendered health oppressions. Drawing on feminist and gender theoretical approaches, and with the health of women and girls as our focus, we seek to extend our understanding of this recursive process by discussing what we call the ‘shaping processes’ of the ‘gender-biology nexus’ which call attention to not only the ‘gender-shaping of biology’ but also the ‘biologic-shaping of gender’. We consider female genital mutilation/cutting as an illustration of this process and conclude by proposing that a framework which attends to *both* the ‘gender-shaping of biology’ *and* the ‘biologic-shaping of gender’ as interweaving processes provides a fruitful approach to theorising the wider health inequalities experienced by women and girls.

## Background

As Raewyn Connell recently explains, ‘in an ontological sense, gender is the way human reproductive bodies enter history, and the way that social process, unfolding through time, deals with biological continuity’ [1, p. 341]. Social relations of gender interact with the biological body to shape the experiences of health of men and women, boys and girls, in numerous ways in manifold geographic contexts worldwide. The aim of this theoretical exposition is to analyse how, within this context, feminist and gender theorists have made biological ‘sex’ and social ‘gender’ legible, with the specific object of identifying lacunae in their expression in a nexus with health. We begin by suggesting that the principal theoretical contribution to date has been to identify how the biological body is shaped by social relations of gender, or what we conceptualise here as ‘the gender-shaping of biology’. We then propose that, notwithstanding calls to re-examine biology in feminist terms [e.g. 2–6], the matter of how the biological body may, by its turn, express and contribute to social gender dynamics in a nexus with health–or what we term the ‘biologic-shaping of gender’–is underexplored. Taking the **‘**gender-biology nexus’ as our object, we put forward a theoretical approach which emphasises two co-constitutive ‘shaping processes’: the ‘gender-shaping of biology’ and the ‘biologic-shaping of gender’ as they operate with respect to the health and health inequalities of girls and women. To explore and illustrate this in a preliminary way, we take the example of female genital mutilation/cutting. In what follows we acknowledge the various meanings given to the terms ‘health’ and ‘illness’, but, given our expository purpose, we generally use the term ‘health’ inclusively to cover both positive and negative dimensions of experience.

## The ‘gender-shaping of biology’

As extensively rehearsed, the sex/gender distinction introduced into feminism in the 1970s [7] had a strong and timely purpose; to challenge the pejoration of the binary script which has fashioned woman’s being as analogous to the biological body, itself conceived as inferior to that of man. This roused the compelling argument that the causes of health/ill-health globally are predominantly social and an effect of women’s inequality within the dominion of men. Of course the argument has never been that ‘biological sex’ and ‘social gender’ bear no relationship, but rather that ‘the aura of naturalness and inevitability that surrounds gender-differentiation’ comes […] from the beliefs people hold about it’, rather than from presumed biological characteristics [7, p. 189]. Even so, research has been, and generally still is, targeted above all towards an examination of the influence of gender as a social factor on women’s bodies and their health [8]. From the 1970s onwards, ground-breaking social science and public health research raised two far-reaching concerns: the generally higher prevalence of ill-health globally of women and girls (compared to men and boys) at the individual and collective levels, and their adverse access to, and treatment in, healthcare settings [e.g. 9, 10]. Anthropologists Nancy Scheper-Hughes and Margaret Lock [11] have encouraged researchers to consider not only the individually experienced ‘body-self’, but also the representational symbolic power of the ‘social body’ to define how nature and culture are thought about in a society–for our interest here, in gendered terms–and the ‘body politic’ which, through healthcare (including lay healing) and other systems such as kinship, regulates both the social body and individual bodies. Stressing that gender itself is global, sociologist Connell [1,12], referred to earlier, has sought to capture the relations of power, production, emotion, and representation that establish the ‘gender order’ and the institutions (e.g. healthcare) that constitute the ‘gender regime’ of a society. She contends that as both agents and objects in reflexive practices, bodies cannot be conceived as *either* biologically *or* socially determined. Here ‘gendered social embodiment’ occurs in a structured interplay with the ‘reproductive arena’ where ‘the reproductive possibilities of human bodies are historicized; that is, given specific social forms’ [13] as both ‘*objects of* social practice and *agents in* social practice’ in a ‘loop, a circuit, linking bodily processes and social structures’ [12, p. 67, emphasis original]. These theoretical contributions, amongst others, have been effective and influential broad steers for a wealth of powerful empirical research on ‘gender and health’ internationally [see, for example, 14–17]. However, while the biological body is clearly a point of reference in these and other theoretical contributions, it is mostly tacit. In Connell’s work, for instance, bodily capacities primarily appear to be ‘a site where something *social* happens’, such as the creation of the categories ‘women’ and ‘men’ [12, p. 68, emphasis added]. Her illustrations of anorexia and HIV transmission [13], for example, address the transformation of bodies in social embodiment, but she does not intend to take up the associated biological processes *in* the body. Recently intersectionality has gained theoretical traction as a counter to universal depictions of the experiences of social groups (such as women), pointing to matrices of domination that arise from complex interactions of other social structures such as age, race, class, and citizenship with gender [18]. For example, with reference to global health, Anuj Kapilashrami and Olena Hankivsky [19, p. 2,589] have recently argued that an intersectional approach goes beyond the examination of what they identify as individual factors, such as biology, socioeconomic status, sex, and gender, to explore the impact that interactions among these factors have upon health in a specific context. As they argue, this advances understanding of health inequalities by drawing attention to differences amongst what tend to be seen as relatively homogenous population groups, such as ‘women’, and by highlighting the interacting influence of different ‘multiple sites and levels of power’, such as laws, institutions, and structures of discrimination like sexism on health [19, p. 2,589]. Yet, significant though their points are, and although referring to the interacting role of biology, their attention in illustrations of cardiovascular disease and migration is on the influence of interacting *social* factors with the body. Also taking an intersectional approach, but with a thoroughgoing focus on gender, Kristen Springer and colleagues justly question the positing of sex and gender as distinct domains, explaining instead that ‘the vast majority of male-female health differences are due to the effects of the irreducibility of entangled phenomena of “sex/gender” and therefore that this entanglement should be theorized, modeled, and assumed until proven otherwise’ [20, p. 1,818]. Again, the foremost concern is with the ‘material effects on the body’ of ‘gendered life experiences’ as they ‘*show up’* in ‘biologically based “sex differences”’ [20, p. 1,818, our emphasis]. They cite existing research on matters such as the effects of social interaction and status differentials on neuroendocrine function and psychosocial stress on cardiovascular disease, but they do not intend to detail the biological processes that may be at work.

What we refer to as ‘gender-shaping’ also underlines psychosocial stress research. Often taking its cue from endocrinologist Hans Selye’s [21, p. 692] definition of stress as ‘the non-specific response of the body to any demand made upon it’ (such as emotional upsets on processes such as blood pressure and body temperature), research has addressed the effects (implying stress arousal) of gendered life and working conditions in the biological body. For example, Marianne Frankenhaeuser and colleagues [e.g. 22] have researched the importance of gendered conditions in unpaid work for the differences in stress hormone response between men and women in white-collar occupations. In her influential depiction of ‘embodiment’, social epidemiologist Nancy Krieger [23, p. 350] explores what bodies tell us about lives by the marks left on them by the body politic through, for instance, food insecurity, economic and social deprivation. To depict how biological sex and social gender are, ‘inextricably woven’, she introduced (with Sally Zierler), the lexicon ‘biologic expression of gender’ to characterise the incorporation of social expressions of gender into the body–such as the effects of underfunding of girls’ athletic programs on ‘body build and exercise patterns’ [24, p.42–43] – and the companion concept, the ‘gendered expression of biology’ ‘to show ‘how biologic processes influence gender roles, relations, and conditions’ (such as when the ability to get pregnant is used to restrict women’s employment in typically male and well-paid jobs, even when less well-paid jobs can be more hazardous to health) [24, p. 41]. Here the focus is on biological *expression*, or how our understandings of the biological body are filtered through a gender lens. Subsequently Krieger [25] has drawn attention to the potentially synergistic relationship between what she dubs ‘sex-linked biology’ and ‘gender relations’ in health outcomes. The former depicts the reproductive system, including chromosomal sex, secondary sex characteristics, pregnancy, and menopause. Her proposition that ‘sex-linked biological characteristics can, in some cases, contribute to or amplify gender differentials in health’ [25, p. 653] is instructive. Her examples, such as women’s higher exposure to intimate partner violence–where ‘sex-linked-biology’ is set out as a determinant of strength and stamina, in interaction with ‘gender relations’, such as men’s greater likelihood of using physical violence–are astute, but it is not her goal to explore the actual biological processes at work.

This summary, which for reasons of space cannot do justice to the now sizeable body of writing from gender and feminist thinkers on women’s health within the social sciences, has highlighted how enlightening research on what we refer to as the ‘gender-shaping’ of the biological body has been. However, in this loosely grouped corpus of research, biology has not so much been ignored as left tacit; more tacit, we would argue, than it should be if we are to move towards a more comprehensive understanding of ongoing cycles of women’s health oppressions. In a somewhat separate body of writing, feminist biologists have (as we would expect) given biology a more visible analytic presence. For example, Anne Fausto-Sterling [26,27] deftly explores the interweaving of bodies, disorder and culture under the rubric of ‘life course systems theory’/’dynamic systems theory’. She observes that since social experience produces new biosocial formations, ‘nothing in the body’ is ‘permanent and unchanging’ [28, p. 63]. She rightly argues that temporal changes draw attention to alterations both in individual biological bodies as they grow and age and the transformation of social groups as experiences of earlier generations are embodied in offspring. For example, in an analysis of the skeletal system and osteoporosis, she conjectures that a complex of factors, including physical exercise, diet, drugs, hormones, and biomechanical effects on bone formation interact through the lifecycle to influence bone density and fractures, negatively affecting more women than men. She explicitly acknowledges that we know relatively little scientifically about how these processes and mechanisms occur, but emphasises that they transpire within ‘the experiences of growing, living, and dying in particular cultures and historical periods and under different regimens of social gender’ [26, p. 1,510]. She hypothesizes, for instance, that women’s more frequent dieting to lose weight during their lifetime may contribute to lower peak bone density in adulthood compared to men and hence to fractures. As this indicates, her focus is squarely upon the ‘gender-shaping’ of biology. This is further illustrated through her example [29] of the facility to choose from amongst the social features of gender to embed new bodily habits, such as the capacity, through practice, to alter voice register, tonality and cadence to correspond with that of a typical man or woman and the embodiment of this new habit in the sensorimotor (neuromuscular) system. In a landmark analysis, biologist Lynda Birke chastens fellow feminists for conceptualising the body as ‘the malleable surface of an internally stable corporeality’ [2, p. 137]. Following neuroscientist Steven Rose [30], she argues that although bodies are ‘self-organising and self-determining’ and sometimes ‘outside of our willed control’ [2, p. 169, p. 85], we should conceptualize them not as ‘simply *being*, but *becoming*’ in two-way processes throughout our lives [31, p. 45, emphasis in original]. She guides us very effectively to the fleshy, material body, but, again, we are primarily led towards what we call the ‘gender-shaping of biology’ through changes within the body resulting from social engagement [6].

Clearly the work of feminist biologists is very important. But we still have some way to go if we are to move beyond the analysis of gendered narratives and representations to grasp empirical data *about* the body which, as Margaret Lock and Vinh-Kim Nguyen recently put it, remain black-boxed, obscuring ‘the pernicious, embodied and long-term consequences of social inequalities’ [32, p. 329]. As argued more generally by Thomas Lemke [33, p. 87], amongst others, there is hesitancy amongst many feminists to engage directly with ‘biological data and corporeal materiality of the body’. This hesitancy is explained by the understandable desire to shun the hoary and truculent patriarchal equation of women and girls with a defective biology which has justified women’s inequality through time [8]. Thus it is to some extent understandable that ‘feminist-biologists’ (as we conceptualise them) and other researchers we have discussed seem to grapple primarily with how social processes (variously conceptualised) become embodied and (potentially) generate change in the biological body–itself a thorny, and certainly important, matter–to the relative neglect of the even bristlier and challenging concern of the interacting role that biological changes themselves might play in *shaping gender* in the nexus with health. But, as we now go on to argue, further steps are needed to develop a theoretical framework that tightens up the ‘gender-biology nexus’ in relation to health.

## The ‘biologic-shaping of gender’

**Figure 1.**
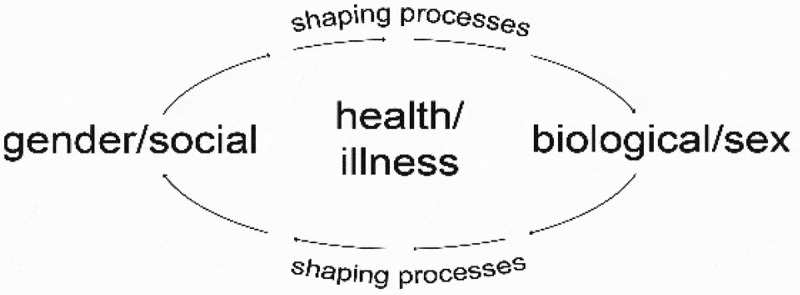
Shaping processes of the ‘gender-biology nexus’.

Though our conceptualization of the ‘gender-shaping of biology’ resonates with present ways of thinking (as described above), the ‘biologic-shaping of gender’ is outwardly less obvious in its meaning. It is therefore important to emphasise that we are not saying that biology has a *determining* role, but rather that cyclical and highly complex ‘*shaping processes’* are likely to be in play whereby biological changes–which have themselves been ‘gender-shaped’ (in the manner depicted by the existing research as discussed)–recursively shape women’s gender-related experiences of health (‘the biologic-shaping of gender’). Hence it should also be noted that we are not suggesting, or intending to identify, a linear ‘input-output’ model whereby the ‘inputs’ of socially gendered experiences generate biological changes which then ‘output’ to effect gendered health experiences anew, but rather an imbricated and recursive process. This process is represented diagrammatically in the Figure 1.

With the advent of ‘new materialist’ feminism [e.g. 6, 34, 35] over roughly the last decade, attention has turned more directly to the materiality of the body as ‘itself an active, sometimes recalcitrant, force’ [34, p. 4]. Samantha Frost [36, p. 71; 37], for example, argues that if feminists wish to grasp the interaction of culture and biology as ‘complex, recursive, and multi-linear’ they must ‘acknowledge that matter and biology are active in their own right’. Humans, as Frost [38] relates, are ‘biocultural’ beings, or, as Karen Barad [35] puts it, formed by ‘naturalcultural’ practices. The living human body comprises a multitude of complex biological processes which bridge the inner body systems with the outer social and gendered context, for example, through perception and cognition. As Frost [38, p.75–76] argues, bodies are responsive to their environments and ‘quite literally rebuild themselves, constantly, in response to the molecular constituents of their habitats’. But they are not identical to their habitats since each body has been formed by its earlier biological and cultural (biocultural) interchanges as well as those of previous generations. For instance, research suggests that epigenetic processes may act as a channel through which social environmental influences affect the body by changing gene expressions (the phenotype) without changing the underlying DNA sequence (the genotype). Epigenetic changes may thus alter gene expressions and modify disease susceptibility in various ways through changes in the epigenome [39] which manifest in material physical form. Thus environmental epigenetics highlights not only the making and remaking of bodies by their environments, but also that bodies are, as Julie Guthman and Becky Mansfield argue, ‘always active in their own remaking’ [40, p. 499]. Recognising that bodies and social/material environments develop in relation to each other destabilises the conventionally conceived social/biology border and draws attention to biological plasticity [41]. Thus the body’s external environments do not sit beyond it, but ‘are themselves partly a consequence of the organism itself as it produces and consumes the conditions of its own existence’ [42, p. 108].

Although this way of thinking is gaining recognition, as Jörg Niewöhner and Margaret Lock [43] instruct, there is a dearth of empirically-informed research in the health field to illustrate just how the biological body may be actively involved in this process. This is notably the case with regard to feminist work on health. As an illustration of how the processes by which the biological body might not only be *shaped by* gender but may itself, by turn, have a role in *shaping* women’s experience of health/ill-health, we take female genital mutilation/cutting (FGM/C) as a case example to begin to examine the body’s biological systems and health inequality. Given the state of current scientific knowledge, this case is offered in a preliminary and tentative fashion.

## The case of FGM/C

Identified by the United Nations as a human rights violation affecting girls and women worldwide, FGM/C is especially concentrated in a swath of countries from the Atlantic coast to the Horn of Africa, in areas of the Middle East, and in some countries of Asia. The WHO defines the practice as comprising ‘all procedures that involve partial or total removal of the external female genitalia, or other injury to the female genital organs for non-medical reasons’ [44]. By recognising that ‘FGM is an act that cuts away equality’ [45], the most recent UN-sponsored International Day of Zero Tolerance 2018 underscored the association of FGM and gender inequality. Worldwide, in countries where it is prevalent, 200 million girls and women alive today have been cut, with 3.2 million cut annually [45–48]. Prevalence varies considerably across countries. Secular trend analysis shows some significant shifts downwards in prevalence over the last twenty to thirty years in some regions, such as East Africa, which according to Demographic Health Survey (DHS) data, saw a reduction in prevalence from 71.4% in 1995 to 8.0% in 2016 [49]. However, UNFPA [47] predicts (also based on DHS data) that due to underlying population growth in girls under age 25, the number of women affected will increase significantly by 2030 in countries where FGM/C is prevalent.

FGM/C is not only a practice, traceable back thousands of years, but also an object of political debate within contemporary feminism and beyond [e.g. 50], making it in Hilary Burrage’s [51] words, a moral maze. UNICEF, for example, has employed both the more politically neutral FGM/C (female genital cutting) and FGM [46,52]. Since we cannot do justice to political debates here, which, although important, are not essential to our purpose, we opt to use the broader term FGM/C. FGM/C is an expression of gender inequality and a form of violent abuse within patriarchal societies past and present [see e.g. 51, 53]. FGM/C’s persistence is often associated with entrenched socio-cultural norms. As a cultural and political marker of inside/outsider status for girls and women, it often symbolises cleanliness, purity, an appropriate embodied femininity and entry into womanhood and is seen to improve fertility and marriageability [51,54,55]. Social exclusion, shame and stigma often result if a girl is not cut [50,52,56]. Associations are often drawn between FGM/C and the Islam since it is well-established in many predominantly Islamic societies (such as in sub-Saharan Africa), yet not all Islamic groups engage in the practice while many non Islamic groups do (it is practised amongst the Christian and Jewish faiths, for example). As Burrage [51] relates, FGM/C is axiomatic to no world religion, yet in various times and place various religious faiths have practised it and patriarchal religions arguably create the milieu which allow the practice to continue.

Although the genito-urinary effects of FGM/C, such as effects on sensibility and sexual pleasure, painful neuromas, micturition difficulties, menstrual, and obstetric complications are fairly well-documented [e.g. 57, 58], in-depth studies of how these complications are embodied and experienced throughout the lives of women are few in number, undoubtedly because of the not inconsiderable practical challenge of conducting research on the matter. Long-term bodily consequences of FGM/C may extend beyond the reproductive system, involving, for instance, intestine and urinary bladder dysfunction and long-term pain and complications [59], as well as somatic complaints; that is, symptoms with no identifiable organic cause, such as aches and pains, and also significant mental health problems, including depression, anxiety, and PTSD [60–62].

To refer back to our Figure 1, throughout our discussion thus far we have focused primarily on one facet of the ‘shaping process’ within the ‘gender-biology nexus’; namely, the ‘gender-shaping of biology’. In the reciprocal process of ‘biologic-shaping of gender’ we attend to how the experience of women and girls may *alter* in complex embodied interactions with biological changes in the body. By definition, when referring to *female* genital mutilation/cutting, it is important that we include ‘sex’ because only the biological sex organs of girls and women i.e. the vulva (clitoris, labia majora, labia minora) are exposed to trauma. While it can be noted that male circumcision (cutting of the prepuce, or foreskin) can also carry health risks (though these are not high) such as haemorrhage and bleeding and erectile dysfunction [63], and that some argue that we should problematise male circumcisions as a routine practice and its association with understandings of the male body and masculinity [64], this is not addressed here as our focus is on women and girls.

Though not referring to FGM/C, Jörg Niewöhner and Margaret Lock argue that bodily sensation and experience is ‘in part *formed by* the material body, itself *contingent on* evolutionary, environmental, social and individual variables’ [43, p. 684, our emphases]. The consequences of these ‘variables’, as Niewöhner and Lock express it, are illustrated in research by Anke Köbach and colleagues [60] with women in Jijiga, the capital of the Somali region of Ethiopia where FGM/C has been widespread. Their analysis is based on a convenience sample (without a control group) and comprises self-reported information gleaned from women in interview (with clinical psychologists) about FGM/C, including experience of the cutting, subsequent short and long-term physical complications, and validated measures of PTSD and other mental health problems. From their analysis the authors identified associations between the most severe kinds of cutting (types II and III) and psychopathological symptoms in adulthood, especially vulnerability to PTSD and shutdown dissociation. They also found higher hair cortisol concentrations (an indicator of hormone response to stress) in women who experienced FGM/C before their first year of age or had more severe forms of FGM compared to rest of the women, which indicates long-term neuroendocrinological consequences of FGM and trauma in general on the central stress system (the hypothalamic-pituitary-adrenal axis, or HPA). Since the HPA axis genes play an important role in regulating the impact of social and environmental stress, Köbach et al. draw attention to the possibility that the trauma from experiencing cutting may have epigenetic effects. That traumas during a critical age period of epigenetic plasticity in early life (as Köbach et al.’s [60] respondents’ experienced) may lead to epigenetic processes is suggested by animal studies [65] and has been proposed as a framework for epigenetic modifications in the biological integration of socioeconomic factors during life. Research indicates that early egregious trauma (such as abuse in childhood and other sorts of early-life stress among humans) may lead to dysregulation of the HPA axis and later life mental ill health [66] as well as other health problems, such as cancer and cardiovascular disease [e.g. 67–69]. Thus we can situate, albeit tentatively (since, as noted, research is very limited at present), findings about FGM/C within the hypothesized associations between stress-induced epigenetic modifications located in early stressful life events during childhood and later life health inequalities in the manner suggested as possible for socio-economic differentials [see e.g. 68, 70]. In our case illustration, possible epigenetic effects reveal that the ‘gender-shaping of biology’ (taking FGM/C to be the effect of women’s environmental and social inequality) appears to entangle with neuroendocrinological changes which ‘biologically-shape’ (but do not determine) the health of girls and women exposed to FGM/C, which can be conceptualised as a form of gendered health inequality. To explore this ‘biologic-shaping of gender’ in relation to FGM/C further, we draw now on the work of Gillian Einstein [71,72], a biologist with a doctorate in neuroanatomy, who explores the neurobiological repercussions of FGM/C from a feminist perspective.

Focusing on FGM/C type III (infibulation, excision of the external genitalia with closure of the introitus) [62], Einstein proposes that cutting of the efferents and afferents (nerve circuits) carried in the pudental, pelvic and hypogastric regions may affect the rest of the body via the central nervous system (CNS) which, along with others [e.g. 73], she describes as ‘sensitive and malleable’ [72, p. 171]. She takes FGM/C’s effects not in isolation and as affecting one part of the body (the reproductive system), but as ‘owned by the entire body, or embodied through the interconnections of all body systems and the environment’ [72, p. 158]. In an expressly speculative analysis she suggests that since the tissue of the vulva is highly innervated, cutting the nerves which supply the skin and muscle will affect the feed-back processes of the central nervous system and rouse long-lasting, body-wide effects such as referred sensations, including pain (referred sensation means a sensation perceived at another location than the site of the stimuli causing the sensation). The spinal cord and brain may respond to cutting with reorganization (‘rewiring’) of neural circuits by referred sensations. The neurological tissues can react to bodily losses akin to the way in which, upon the amputation of a leg, a person may still feel the sensation of parts of the lost leg or feelings of pain in the lost leg–a phenomenon called phantom sensation or phantom pain. Einstein [71] suggests similarly that women exposed to FGM/C may experience phantom sensations or clitoral pain.

Extrapolating from Einstein’s arguments, while the (new) biological changes to the body may *shape* physical sensations after having been cut, we would not expect them to *determine* sensate experience in any simple or universal way because women’s interpretations of and responses to biological change are situated in time and place and therefore formed by local expectations and practices. To deploy anthropologist Margaret Lock’s [74] well-known concept of ‘local biologies’, the shaping processes that we highlight here are contingent and experienced in specific gendered environments. According to Einstein [71,72], it is reasonable to argue that as it is affected by other bodily modifications, the CNS itself ‘plays a role in the embodiment of culture’ [72, p. 155] with potential gendered consequences for both the bodies and minds of women and girls. Thus she proposes that cutting not only makes girls and women resemble their community physically (which is likely to be normatively valued), ‘through its actions on the CNS it inscribes values of comportment and aesthetics’ [71, p. 94]. Thus she relates that FGM/C ‘configures the ways in which a woman carries herself, walks, and experiences the world’ [71, p. 94]. By this we may infer that a new collective and individual mind-body is produced. First-person experiential accounts provide support for this. Waris Dirie [75] and Hibo Wardere [56], for instance, explain how their physical bodies changed after cutting and the horrific pain when urinating and the nightmare of menstrual periods after being cut as young girls. Reflecting back on the impact of biological change on her life as a girl, Wardere laments, ‘no more running, skipping or jumping rope for me’ [56, p. 223]. Similarly, in research by Morison and colleagues [76], Somalis living in London spoke of direct effects of cutting which involved walking and behaving differently to avoid opening up scars. This conjures political scientist Iris Marion Young’s [77] classic discussion of female comportment. Less open than men in gait and stride, Young argues that ‘modalities of female bodily existence’ are rooted in experience of the body as a ‘fragile thing, which must be picked up and coaxed into existence’ [77, p. 39]. Perforce, women who have been cut may realise pain, distress, and constricted physicality, but as this usually is all they and those around them know, over time and through generations, as Einstein explains, experiential changes may become ‘instantiated as the “normal” (and perhaps, desirable) body’ [72, p. 151; see also, 78] and hence part of the experience of womanhood [56,75]. Research with Somali-Canadian women, for example, has shown that wide-scale bodily pain and discomfort can be brushed-aside as normal-natural as women exhibit resilience through the desire not to let pain attain power over their lives [71,72,78]. Nevertheless, as Johansen [79] explores, the pain of infibulation has lasting effects, which Somali refugee women in her Norwegian study spoke of as ‘embodied memory’ carried with them as a burden and sense of loss. This then points to how shaping processes; the intertwined ‘gender-shaping of biology’ and consequent ‘biologic-shaping of gender’ through time, may produce a new collective and individual mind-body, as noted earlier.

To return explicitly to our Figure 1, while the origins of FGM/C are indisputably social and seated in localised social relations of gender (‘gender-shaping of biology’), they may effect complex and perhaps far-reaching changes in the material biological body. The body becomes other than what it once was (or could have been); it is altered. Through our illustration, we have sought to open up black-boxed data about the body which obscures the harmful embodied and long-term consequences of social inequalities [43] by bringing to light the epigenetic and neurobiological processes through which changes may occur. These bodily changes by *their* turn entwine with (but do not determine) women’s individual and collectively gendered bodily expressions and experiences (the ‘biologic-shaping of gender’) which are unlikely to be universal, but rather to vary by time and place. It is important to stress that by this argument we do not intend to say that the biological and the social are one and the same, collapsed into one another or, as noted earlier, that a linear ‘input-output’ process is in play, but rather that gender-suffused social milieu–which encompass, for example, the health, life and experiences of our illustration–become sedimented (but not ineludibly fixed) in bodily practices which concern women’s health as individual and collective lives evolve in time.

## Implications for policy

As remarked upon at the start, it is important that theoretical frameworks and conceptual tools are subject to ongoing critical analysis because they shape research outcomes by guiding research pathways. A thoroughgoing analysis of the global production of women’s health inequality depends on a comprehensive theorization of how social relations of gender and the biological body mutually inform each other in local contexts. To pick up on the recent statement referred to earlier from UN Women [45] that ‘FGM is an act that cuts away equality’, we argue that a comprehensive understanding of what this means for women’s health calls for us to go beyond the common concern with how social and cultural practices shape the biological body–important though this, of course, is – to also attend to the recursive effects of the biological changes themselves on women’s social lives and lived bodily experiences. Yet we very quickly reach the limits of our empirical knowledge when we try to develop this more comprehensive approach. A primary reason for this is the distinct lack of interdisciplinary research. While feminist and gender theorists have begun to explore the biological substance of the body as active, rather than passive, matter [such as in materialist feminism e.g. 35,38], they have not directly engaged with health experiences associated with inequality for women and girls. Even in the field of FGM/C, for example, there is a paucity of in-depth qualitative research exploring embodied experience. Thus a recommendation made here, which accords more generally with those made in the wider context of women’s health [e.g. 80,81], is that research funding bodies and institutions recognise the value of interdisciplinary theoretical and empirical research in the field commonly known as ‘gender and health’ that addresses not only the ‘gender-shaping of biology’ but also the ‘biologic-shaping of gender’ and which avoids essentialist and reductivist thinking.

## Conclusion

In this theoretical paper we have sought to explore how social relations of gender interrelate with the biological body to shape the experience of health in ways that may generate inequality for women and girls. Specifically we have analysed how feminist and gender theorists have made biological ‘sex’ and social ‘gender’ legible, with the specific object of identifying gaps in their expression in a nexus with health. We have argued that, to date, most attention has been directed to what we call the ‘gender-shaping of biology’ to the relative neglect of the co-constitutive role that biological changes *themselves*–what we dub the ‘biologic-shaping of gender’–may play in ongoing cycles of gendered health inequality. FGM/C has been taken to explore in a preliminary way how these ‘shaping processes’ may occur. It is recognised, however, that we are limited in our capacity to fully substantiate what we conceptualise as the shaping processes of the ‘gender-biology nexus’ (focusing on health and illness) at the present due to lack of research. In order for this to progress, we suggest that far more interdisciplinary research between social scientists, including gender theorists, and biological and health scientists is needed.

## Paper context

Analyses of the relationship between social and biological factors in the production of women’s health inequality have focused predominantly on how social factors become embodied to the relative neglect of the reciprocal role played by the biological body in shaping its social world. Drawing on feminist and gender theory we develop a novel theoretical approach which attends not only to the ‘gender-shaping of biology’ but also to the ‘biologic-shaping of gender’ as interweaving processes.
